# Advancing Drug Repurposing for Rheumatoid Arthritis: Integrating Protein–Protein Interaction, Molecular Docking, and Dynamics Simulations for Targeted Therapeutic Approaches

**DOI:** 10.3390/cimb47121039

**Published:** 2025-12-12

**Authors:** Krishna Swaroop Akey, Bharat Kumar Reddy Sanapalli, Dilep Kumar Sigalapalli, Ramya Tokala, Vidyasrilekha Sanapalli

**Affiliations:** 1 Department of Pharmaceutical Chemistry, Shri Vile Parle Kelavani Mandal’s College of Pharmacy, Shirpur 425421, MH, India; krishnaswaroop1997@gmail.com; 2 Department of Pharmacology, School of Pharmacy & Technology Management, SVKM’s Narsee Monjee Institute of Management Studies (NMIMS) Deemed-to-be-University, Jadcherla 509301, TS, India; bharathsanapalli@yahoo.in; 3 Department of Biochemistry, University of Washington, Seattle, WA 98195, USA; dilep@uw.edu; 4 Department of Radiology, Athinoula A. Martinos Center for Biomedical Imaging, Massachusetts General Hospital and Harvard Medical School, Charlestown, MA 02129, USA; 5 Department of Pharmaceutical Chemistry, School of Pharmacy & Technology Management, SVKM’s Narsee Monjee Institute of Management Studies (NMIMS) Deemed-to-be-University, Jadcherla 509301, TS, India

**Keywords:** rheumatoid arthritis, drug repurposing, network pharmacology, molecular docking, MD simulation

## Abstract

**Background**: Rheumatoid arthritis (RA) is a systemic chronic inflammatory autoimmune disease causing progressive joint destruction, resulting in significant morbidity and increased mortality. Despite advances in treatment, current pharmacological options, including NSAIDs, DMARDs, and biological agents, have limitations in tissue repair and can lead to severe side effects. **Objectives**: This study aims to explore drug repurposing as a viable approach to identify novel therapeutic agents for RA by utilizing existing FDA-approved drugs. **Methods**: We applied an integrated computational strategy that uniquely combines network pharmacology with molecular docking and dynamics simulations. The process began with the construction of a protein–protein interaction (PPI) network from 2723 RA-associated genes, which identified five central targets: TNF-α, IL-6, IL-1β, STAT3, and AKT1. We then built protein–drug interaction (PDI) networks by screening 2637 FDA-approved drugs against these targets. Critically, the top candidates from this network analysis were not just docked but were further validated using 100 ns molecular dynamics simulations to thoroughly evaluate binding affinity, complex stability, and interaction dynamics. **Results**: This multi-tiered computational workflow identified Rifampicin, Telmisartan, Danazol, and Pimozide as the most promising repurposing candidates. They demonstrated strong binding affinities and, importantly, formed stable complexes with TNF-α, IL-6, IL-1β, and STAT3, respectively, in dynamic simulations. The key innovation of this study is this sequential funnel approach, which integrates large-scale network data with atomic-level simulation to prioritize high-confidence drug candidates for RA. **Conclusions**: In conclusion, this study highlights the potential of repurposing FDA-approved drugs to target key proteins involved in RA, offering a cost-effective and time-efficient strategy to discover new therapies.

## 1. Introduction

Rheumatoid arthritis (RA) is an autoimmune condition and a systemic chronic inflammatory disease that causes progressive inflammation within the synovial tissues. This persistent inflammatory response eventually destroys the bone and cartilage within the affected joints, resulting in higher mortality, a reduction in lifespan, and progressive disability [1]. The prevalence rates range from 0.5% to 1% across the US population, 0.9% within India, and around 0.16% within the Middle East and North Africa [2,3,4,5,6]. Women are affected by RA up to threefold more frequently than men, with a frequency of roughly 1%. Although the condition can strike any individual at any age, most cases emerge between the ages of 30 and 50 [7,8]. The actual pathophysiology of RA is yet unclear; however, several variables, including genetic, environmental, and immunological ones, have contributed to a greater prevalence of the onset of clinically persistent RA. The two main environmental risk factors for RA are smoking and alcohol consumption, which increase the probability of developing RA by 40 times compared to non-exposure. Both innate and adaptive immunity have a significant impact on the development of RA and constitute the immunological factors. The risk of RA can also be elevated by other variables, including high consumption of sugar, salt, red meat, protein, and iron; low intake of vitamin D and antioxidants; low birth weight; breastfeeding; and place of birth [9]. Numerous genetic investigations have shown that between 30% and 60% of RA cases are caused by genetic factors. Genes such as T cell signaling genes and cytokine promoter genes contribute the majority of genetic factors. These genes include RA-related information. A major genetic component associated with RA is the locus for the human leukocyte antigen (HLA-DRB1) [10]. Individuals who smoke and have HLA-DRB1 are more likely to produce more anti-citrullinated protein antibodies (ACPA), which can eventually result in the development of RA [11]. Thus, ACPA serves an integral part in the pathophysiology of RA.

For the last few decades, there has been an advancement in the better understanding of RA pathogenesis, which ultimately led to the production of several medications for the treatment of RA. However, to date, the present pharmacological therapies, including NSAIDs, disease-modifying anti-rheumatic drugs (DMARDs), and biological agents, have not been able to enhance or repair the injured tissues. Furthermore, the current medications impair the immune system, which can lead to significant adverse effects in patients [12]. In this context, drug repurposing has surfaced as a potentially effective strategy for discovering novel therapy alternatives for RA. There are several benefits associated with drug repurposing, as opposed to conventional drug development, including the ability to reposition currently approved drugs for novel therapeutic indications. It leverages the extensive data on the safety and effectiveness of already authorized medications, potentially accelerating the availability of novel therapies while reducing development costs [13,14,15,16,17]. This strategy is earning attraction within the medical fields, including RA.

The pharmacological management of Rheumatoid Arthritis is structured around a multi-layered approach. The first line of treatment typically involves conventional synthetic disease-modifying antirheumatic drugs (csDMARDs), such as methotrexate, leflunomide, and sulfasalazine. Methotrexate acts as an antifolate and immunosuppressant, while leflunomide inhibits pyrimidine synthesis in activated lymphocytes. For symptom relief, non-steroidal anti-inflammatory drugs (NSAIDs) like naproxen and ibuprofen are used to inhibit cyclooxygenase (COX) enzymes, and glucocorticoids like prednisone provide broad anti-inflammatory and immunosuppressive effects. When conventional therapy is insufficient, treatment escalates to biologic DMARDs (bDMARDs), which target specific components of the immune system [18,19]. This category includes: TNF-α inhibitors (e.g., etanercept, adalimumab, infliximab) that bind and neutralize tumor necrosis factor-alpha. IL-6 receptor antagonists (e.g., tocilizumab, sarilumab) that block the interleukin-6 pathway. IL-1 receptor antagonists (e.g., anakinra) that inhibit interleukin-1. T-cell costimulation blockers (e.g., abatacept) that prevent full T-cell activation by binding to CD80/CD86. B-cell depleters (e.g., rituximab), an anti-CD20 monoclonal antibody that targets and depletes B-cells. A more recent class of approved drugs is the targeted synthetic DMARDs (tsDMARDs), primarily JAK inhibitors like tofacitinib, baricitinib, and upadacitinib. These oral medications work by intracellularly blocking the Janus kinase-signal transducer and activator of transcription (JAK-STAT) signaling pathway, thereby inhibiting the action of multiple inflammatory cytokines. The drug development pipeline remains active, with several agents in clinical trials. These include Bruton’s tyrosine kinase (BTK) inhibitors like fenebrutinib, which blocks B-cell receptor signaling; next-generation JAK inhibitors such as the selective JAK1 inhibitor SHR0302 and the JAK3 inhibitor decernotinib (VX-509); and various p38 MAPK inhibitors like VX-702 and SCIO-469, although this class has shown limited efficacy to date. In pre-clinical studies, researchers are investigating inhibitors targeting other pathways, including PI3K (e.g., GS9901), mTOR (rapamycin), Notch signaling (LY411575), and STAT3 (STA-21). There is also significant interest in epigenetic modifiers, such as DNA methyltransferase (DNMT) inhibitors (azacitidine, decitabine) and histone deacetylase (HDAC) inhibitors (e.g., MI192, CKD-506), which can alter gene expression patterns in immune cells [19,20,21]. Finally, cutting-edge technology therapies are being explored, such as PROTACs designed to degrade pathogenic proteins like JAK; nanoparticles for targeted drug delivery to inflamed joints; and CRISPR-Cas9 gene editing to create smart cells that autonomously release anti-inflammatory drugs [21,22].

Despite this extensive arsenal, RA remains an incurable disease, and treatment follows a pyramid strategy of escalating therapy. All current drugs carry a risk of significant side effects, including immunosuppression, cytopenia, and liver damage. This ongoing challenge of balancing efficacy with toxicity underscores the strategic value of drug repurposing investigating clinically safe drugs from other therapeutic areas as a promising pathway to discover new RA treatments with improved safety profiles.

The motivation for this study stems from the urgent need to identify novel therapeutic agents that can overcome the limitations of current RA treatments. Drug repurposing offers a promising solution by leveraging the safety and efficacy profiles of existing FDA-approved drugs to uncover new therapeutic uses. This approach can significantly reduce the time and cost associated with drug development, providing patients with quicker access to effective treatments. By focusing on the molecular mechanisms underlying RA, particularly the key proteins involved in the disease’s pathogenesis, this study aims to identify existing drugs that can be repurposed to modulate these targets effectively. The comprehensive analysis involving protein–protein interactions, protein–drug interactions, and molecular docking studies ensures a robust and targeted approach to drug discovery. Moreover, incorporating molecular dynamics (MD) simulations will enhance the accuracy and reliability of our findings by providing deeper insights into the stability, flexibility, and binding mechanisms of the drug-target complexes at the atomic level. MD simulations can reveal the dynamic nature of protein–ligand interactions, offering a more comprehensive understanding of the interactions than traditional docking methods alone. This added layer of analysis ensures that the selected drug candidates not only bind effectively to their target proteins but also maintain stability and favorable behavior in a biological context [23,24]. This research aims to identify novel therapeutic options for RA and demonstrates the utility of a specific computational workflow for drug repurposing, a strategy that holds promise for application to other complex diseases, thereby broadening the scope and impact of pharmaceutical research.

## 2. Materials and Methods

### 2.1. In Silico Studies

The research employed a combination of online and offline bioinformatics tools for comprehensive in silico analysis. Cytoscape 3.10.1 was utilized for constructing biological networks, specifically protein–protein interaction (PPI) networks, and for drug repurposing through protein–drug interaction networks [25]. For online tools, the PubChem Database was accessed to obtain ligand structures, while the Protein Data Bank (PDB) provided access to high-resolution protein crystal structures [26,27]. Offline tools included Marvin Sketch, which was used for ligand preparation, and Swiss PDBViewer, used for protein structure preparation. Docking studies were conducted using PyRx 0.8 to simulate and analyze protein–ligand interactions [28]. Additionally, Discovery Studio Visualizer client 2020 was utilized for detailed visualization of 2D interactions between proteins and ligands [29]. This integrated approach, leveraging both online databases and specialized software, enabled a robust in silico analysis to identify and evaluate potential therapeutic targets and interactions relevant to RA.

#### 2.1.1. Collection of RA Targets from the DisGeNet Webserver

The targets associated with RA were systematically collected from the DisGeNet web server using specific identifiers and classifications (https://www.disgenet.org/search (accessed on 20 July 2024)). The disease was queried with the name RA and its unique identifier, UMLS CUI: C0003873. This condition is classified under multiple categories: Skin and Connective Tissue Diseases, Musculoskeletal Diseases, and Immune System Diseases, according to the MeSH (Medical Subject Headings) classification system, with the MeSH identifier D001172. Additionally, the disease is referenced in the OMIM database with the entry number 180300 and is categorized as a disease or syndrome under the semantic type. RA is also noted for its phenotypic abnormality, specifically an abnormality of the skeletal system, and is defined in the Disease Ontology as a disease of the anatomical entity. Utilizing these comprehensive classifications and identifiers, a total of 2723 gene-disease associations were retrieved from DisGeNet. These targets form the basis for subsequent PPI network analyses, facilitating a deeper understanding of the molecular mechanisms and potential therapeutic targets for RA [30,31,32,33].

#### 2.1.2. Construction of PPI Network Through Cytoscape 3.10.1

In this study, Cytoscape 3.10.1 was employed as a powerful tool for constructing a comprehensive PPI network using data retrieved from DisGeNet. A total of 2723 targets associated with RA were integrated into Cytoscape, utilizing information sourced from the STRING and STITCH plugins. The STRING protein query option facilitated the creation of an intricate network, providing insights into the complex interplay among these targets at the protein level [34,35]. The CytoNCA plugin was instrumental in the in-depth analysis of the constructed PPI network. CytoNCA enabled the assessment of network topology metrics such as degree centrality, betweenness centrality, and closeness centrality. These metrics are crucial in identifying highly connected and influential nodes within the network, often indicative of key proteins implicated in the pathogenesis of RA. Based on the results obtained from CytoNCA analysis, the top 5 targets were identified as pivotal candidates associated with RA. These targets exhibit significant centrality measures, suggesting their potential importance as therapeutic targets for drug design and development to combat this autoimmune disorder [36,37]. The findings and analyses were documented and saved in CSV format, ensuring systematic recording and further utilization of the identified top targets for subsequent protein–drug interaction studies. This approach lays a solid foundation for advancing our understanding of the molecular mechanisms underlying RA and facilitating the discovery of novel therapeutic interventions.

#### 2.1.3. Construction of Protein–Drug Interaction (PDI) Network Through Cytoscape 3.10.1

In this study, we utilized Cytoscape 3.10.1 to construct protein–drug interaction (PDI) networks by integrating 2637 FDA-approved drugs obtained from the DrugBank database. Building upon the previously identified top 5 targets from the PPI network analysis, we proceeded to create specific PDI networks for each of these targets [38,39,40]. The construction of these PDI networks involved querying the STRING compound database within Cytoscape, which enabled the visualization and analysis of interactions between the selected targets and FDA-approved drugs. Using the CytoNCA plugin, we conducted network analysis focusing on key metrics such as degree centrality, betweenness centrality, and closeness centrality [41,42]. These metrics were instrumental in identifying drugs that exhibit significant interactions with the target proteins, thereby highlighting potential candidates for further investigation in drug repurposing strategies [39]. The results of the network analyses, including all drugs interacting with the top 5 targets, were systematically recorded, and saved in CSV file format. This approach facilitates drug repurposing by leveraging existing FDA-approved drugs and provides insights into novel therapeutic approaches for treating RA. The identified drugs and their interactions with the targets will be further investigated through molecular docking studies, aiming to elucidate the binding modes and potential efficacy of these drugs in modulating the biological activities of the target proteins associated with RA.

#### 2.1.4. Molecular Docking for Top Targets and Interacting Drugs

Molecular docking represents a crucial step in modern drug discovery, playing a pivotal role in predicting and optimizing interactions between small molecule ligands and target proteins. This approach is integral to our research aimed at exploring drug repurposing strategies for RA, focusing on the identified top targets from earlier protein–protein and protein–drug interaction network analyses.

##### Preparation of Proteins

To prepare the target proteins for molecular docking, we employed the Swiss Protein Data Bank Viewer (SPDBV). This offline tool facilitated the retrieval and initial analysis of protein structures downloaded from the PDB. The proteins were processed by removing co-crystallized ligands, applying energy minimization to optimize their conformations, and validating their structural integrity using Ramachandran plot analysis [40].

##### Identification of Binding Sites

Active binding sites within the catalytic pockets of each target protein were identified using the online tool Protein–Ligand Interaction Profile (PLIP). This step was essential for pinpointing regions on the protein surface likely to interact with small molecule ligands [42,43].

##### Preparation of Ligands

We utilized the results from protein–drug interaction networks generated in Cytoscape for the ligand preparation, focusing on 2637 FDA-approved drugs. Specific drugs interacting with each target protein were selected and retrieved from the PubChem Database. Using the Marvin Sketch online tool, these drugs were converted into 3D structures and optimized to identify the lowest energy conformers using MMFF94 force field calculations [44].

##### Molecular Docking

Molecular docking simulations were conducted using PyRx 0.8, an intuitive virtual screening tool tailored for docking studies. PyRx, developed in Python, offers a user-friendly interface and employs Autodock Vina (PyRx 0.8) for efficient ligand docking. The docking process involved preparing the protein target as a macromolecule, optimizing ligand energy states, generating receptor grids to define binding sites, and conducting docking simulations for each ligand-protein pair. The molecular docking results were analyzed to predict the binding affinity and interaction modes between the selected drugs and their respective protein targets. Docking scores and binding conformations were meticulously evaluated and recorded in CSV format for further quantitative and qualitative analysis. This comprehensive approach integrates computational techniques with molecular biology, paving the way for the identification of potential therapeutic candidates and contributing to the ongoing efforts in drug discovery and development for RA [39,41,45,46].

#### 2.1.5. MD Simulation

To assess the stability and behavior of the protein–ligand complexes, we, as part of our research team, employed MD simulations, a powerful tool for studying the dynamic interactions between ligands and their target proteins. The stability of these complexes is crucial for understanding how potential inhibitors might block receptor activity. MD simulations provide insights into the conformational landscape of protein–ligand complexes at a specific temperature, allowing us to observe the changes in the structure and dynamics of these complexes over time. Our team conducted the MD simulations to evaluate the stability, function, and binding free energy of the protein–ligand complexes formed during the molecular docking process. This approach is vital for predicting the effectiveness of small molecule inhibitors. By comparing the behavior of the five target-ligand complexes, we identified key differences that may influence the efficacy of these inhibitors in targeting RA. The simulation results provided valuable information about the binding stability of each complex, guiding us in prioritizing the most promising drug candidates for further experimental validation. The docked complexes, derived from PyRx docking studies, served as the starting point for the MD simulations. These simulations were performed using the Desmond module of the Schrodinger Suite 2021-4, employing the OPLS3e force field to ensure accurate modeling of molecular interactions. Water molecules were modeled using the TIP4P model and placed in an orthorhombic box, with a 10 Å buffer zone between the protein atoms and the box sides to explicitly solve the complexes. System minimization was performed using the OPLS3e force field, employing 200 steps of steepest descent followed by 1000 steps of the conjugate gradient method until a gradient threshold of 25 kcal/mol was reached. Short- and long-range electrostatic interactions were described using the smooth particle mesh Ewald (SPME) method, with a short-range cutoff of 9.0 Å and a long-range electrostatic tolerance of 1 × 10^−9^. Simulations were conducted under isothermal–isobaric conditions (NPT ensemble) at a temperature of 300 K and a pressure of 1 bar. To maintain stability, a Martyna–Tobias–Klein barostat and a Nose–Hoover thermostat was used. The simulations ran for 100 nanoseconds with a time step of 2 fs. The reversible reference system propagator algorithm (RESPA) was employed for integrating bonded, nonbonded, and near nonbonded interactions with varying time steps [39,41,47,48]. The MD simulation results serve as a crucial step in refining our drug repurposing strategy, allowing for the identification of the most stable and promising inhibitors for the treatment of RA.

## 3. Results and Discussion

### 3.1. PPI Network Analysis Through Cytoscape 3.10.1

**Figure 1:**
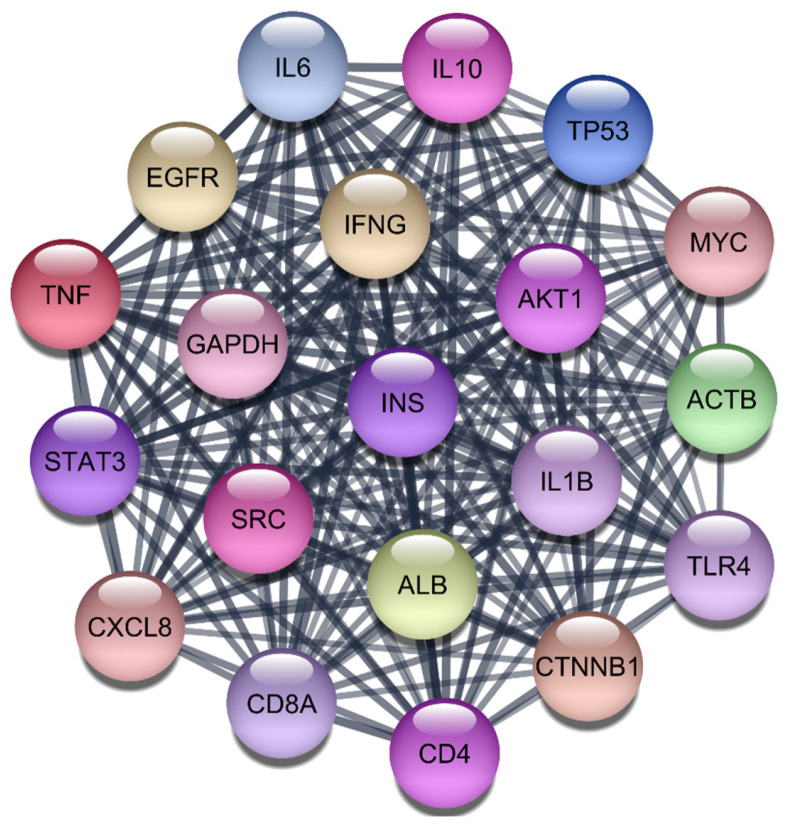
Top 20 Proteins in the PPI Network.

Study began by retrieving a total of 2723 gene-disease associations related to RA from the DisGeNet webserver. After filtering out redundant entries, we retained 2406 unique genes, which were then imported into the STRING protein query database to construct a comprehensive PPI network. This network consisted of 2406 nodes, each representing a gene or protein, interconnected by 96,477 edges, signifying the interactions between these biomolecules. The constructed PPI network was subjected to rigorous analysis using the CytoNCA plugin within Cytoscape 3.10.1. This plugin allowed us to evaluate the network based on three critical centrality parameters: degree, betweenness, and closeness, using the ‘without weight’ criterion. These parameters are vital for understanding the structural properties of the network and identifying the most influential nodes that play key roles in disease mechanisms. Our analysis yielded a ranked list of the top 20 target genes based on their centrality scores. These results are summarized in Table 1 and Figure 1. Among these, TNF, IL-6, IL-1Beta, AKT1, and STAT3 emerged as the most prominent targets due to their high degree, betweenness, and closeness scores. Specifically, TNF showed the highest degree (913) and betweenness centrality (167,860.9), highlighting its significant role in the network. IL-6 and IL-1Beta also demonstrated substantial influence with degree values of 878 and 802, respectively, along with notable betweenness and closeness scores. AKT1 and STAT3, both critical components in signaling pathways associated with inflammatory responses, were also identified as top targets with degree values of 802 and 679, respectively. These findings underscore the importance of these proteins in the pathogenesis of RA, particularly their involvement in cytokine signaling and inflammatory processes. This multi-step analysis, combining network topology and literature validation, ensured that our selected targets were both statistically significant and biologically relevant. The identified top five targets IL-1Beta, IL-6, TNF-α, AKT1, and STAT3 are crucial for subsequent drug repurposing and molecular docking studies aimed at developing effective therapies for RA.

### 3.2. Protein–Drug Interaction (PDI) Network Analysis for RA Targets

To explore potential therapeutic options for the five selected target genes, we conducted a comprehensive drug repurposing study using 2637 FDA-approved drugs from the STITCH compound query database. These drugs were evaluated in batches of 50 to determine their interactions with each target protein, ultimately generating a biological network to identify molecules capable of direct interaction with the proteins of interest.

A critical consideration when interpreting PDI network results is that this unbiased screen captures interactions based on database evidence, which can include compounds with poor suitability for therapeutic inhibition of intracellular PPIs. For example, endogenous metabolites like lysine and glutamine possess low molecular weights and lack the complexity needed for effective PPI inhibition. Similarly, a compound like formalin has poor lipid solubility and high toxicity, preventing effective cellular entry, while retinoic acid is typically used topically and has pharmacokinetic properties less suited for systemic RA treatment. Therefore, the results of this initial network screen were interpreted with these pharmacological and drug-like filters in mind. The subsequent molecular docking and dynamics simulations served as the critical next step to prioritize compounds with favorable binding modes, molecular size, and potential for cellular bioavailability, which is why such non-viable candidates were deprioritized for further investigation.

#### 3.2.1. Interleukin 1-Beta (IL-1β)

**Figure 2:**
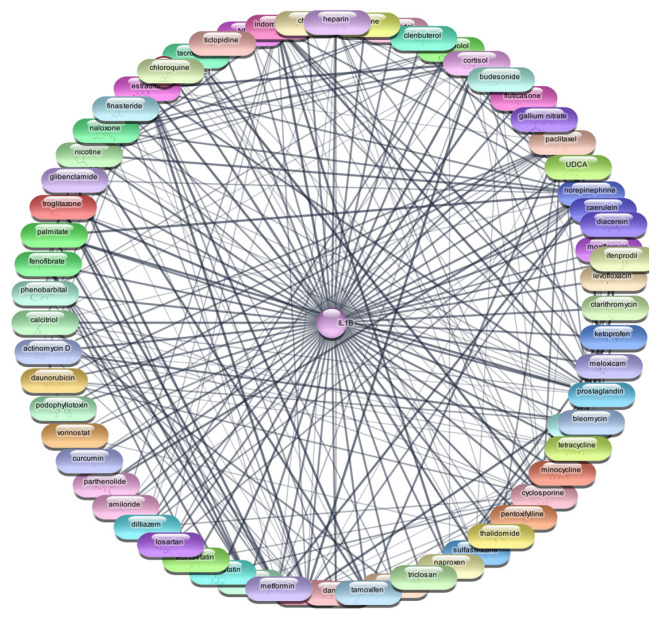
Interaction Network of 65 FDA-Approved Drugs with IL-1β.

**Figure 3:**
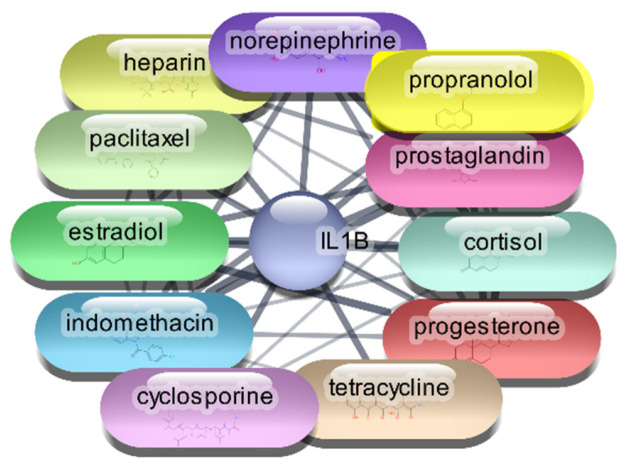
Top 15 FDA-Approved Drugs with Strongest Interactions with IL-1β.

For the IL-1β protein, we screened all 2637 FDA-approved drugs and identified 65 drugs that demonstrated direct interactions with this target. The network analysis for these interactions was performed using Cytoscape, focusing on centrality metrics such as degree, closeness, and betweenness to evaluate the significance and impact of each drug within the network. Table 2, Figure 2 and Figure 3 presents the top 10 drugs interacting with IL-1β, ranked by their degree, closeness, and betweenness centrality scores. These metrics are crucial for understanding how influential a drug is within the network and its potential efficacy as a therapeutic agent.

Cyclosporine emerged as the most significant drug, exhibiting the highest degree (26) and betweenness centrality (0.614679), indicating its potential as a highly influential molecule within the PDI network. Prostaglandin, norepinephrine, and indomethacin also showed strong interactions, with considerable degree and betweenness values, suggesting their relevance in modulating IL-1β activity. The identified drugs were subjected to further analysis through molecular docking studies with the IL-1β protein. This step aimed to validate the predicted interactions and assess the binding affinities of these drugs, providing insights into their potential efficacy as repurposed treatments for RA. This meticulous approach, combining network analysis and molecular docking, enhances our understanding of the therapeutic landscape for RA and identifies promising candidates for drug repurposing.

#### 3.2.2. AKT1 Protein–Drug Interaction Analysis

For the AKT1 protein, we conducted an extensive screening of 2637 FDA-approved drugs to identify potential therapeutic agents. This screening revealed 106 drugs that demonstrated direct interactions with AKT1. To further analyze these interactions, we utilized Cytoscape software, which enabled us to construct and evaluate the protein–drug interaction network. Using centrality metrics such as degree, betweenness, and closeness, we assessed the significance and impact of each drug within the network. These metrics are pivotal for understanding the influence of each drug and its potential efficacy in targeting AKT1. Degree centrality indicates the number of direct connections a drug has within the network, betweenness centrality measures the role of a drug in facilitating interactions between other nodes, and closeness centrality indicates how quickly a drug can interact with other nodes.

**Figure 4:**
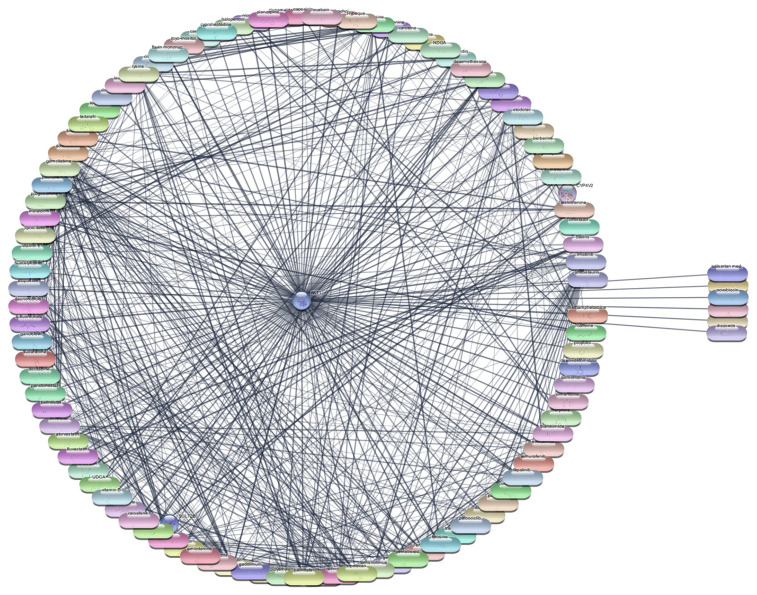
Interaction Network of 106 FDA-Approved Drugs with AKT-1.

**Figure 5:**
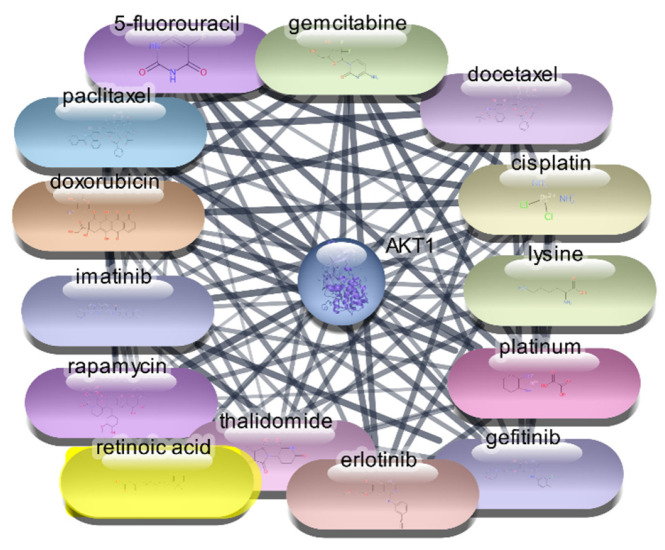
Top 15 FDA-Approved Drugs with Strongest Interactions with AKT-1.

The top 10 drugs interacting with AKT1, ranked by their degree, betweenness, and closeness centrality scores, are presented in Table 3, Figure 4 and Figure 5. These top-ranked drugs are considered the most influential within the network and hold the highest potential as therapeutic agents for modulating AKT1 activity in the context of RA. The protein–drug interaction analysis for AKT1 revealed several FDA-approved drugs with significant potential for therapeutic intervention in RA. Paclitaxel emerged as the top drug, demonstrating the highest degree centrality (36), indicating a strong number of direct interactions within the network. This was followed closely by cisplatin and doxorubicin, both with a degree centrality of 35, highlighting their substantial connectivity. The betweenness centrality metric identified lysine as a critical mediator within the network, possessing the highest score (248.9429), suggesting its pivotal role in bridging interactions between other drugs and the AKT1 protein. Closeness centrality, which measures the average shortest path to all other nodes, was highest for paclitaxel (0.596591), underscoring its efficiency in interacting with the network. Other notable drugs include imatinib, docetaxel, and rapamycin, each displaying robust centrality scores, indicating their potential effectiveness as therapeutic agents targeting AKT1. This comprehensive network analysis highlights these top 10 drugs as prime candidates for further molecular docking studies, aiming to validate their interactions and explore their utility in RA treatment.

#### 3.2.3. STAT3 Protein–Drug Interaction Results

For the STAT3 protein, an extensive screening of 2637 FDA-approved drugs was conducted to identify potential therapeutic agents. This screening identified 49 drugs that demonstrated direct interactions with STAT3. The interactions were further analyzed using Cytoscape software, enabling the construction and evaluation of a protein–drug interaction network. Centrality metrics such as degree, betweenness, and closeness were employed to assess the significance and impact of each drug within the network. Degree centrality reflects the number of direct connections a drug has within the network, betweenness centrality measures a drug’s role in facilitating interactions between other nodes, and closeness centrality indicates how efficiently a drug can interact with other nodes. These metrics are pivotal for understanding the influence of each drug and its potential efficacy in targeting STAT3.

**Figure 6:**
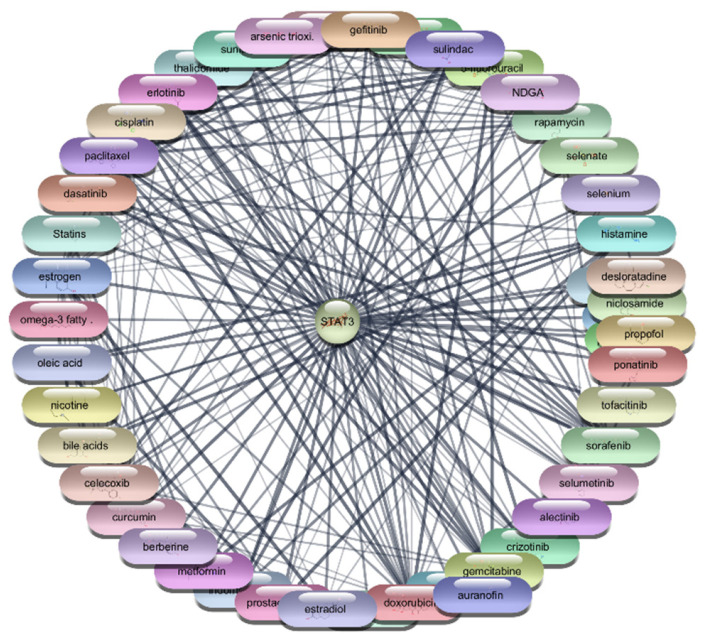
Interaction Network of 106 FDA-Approved Drugs with STAT-3.

**Figure 7:**
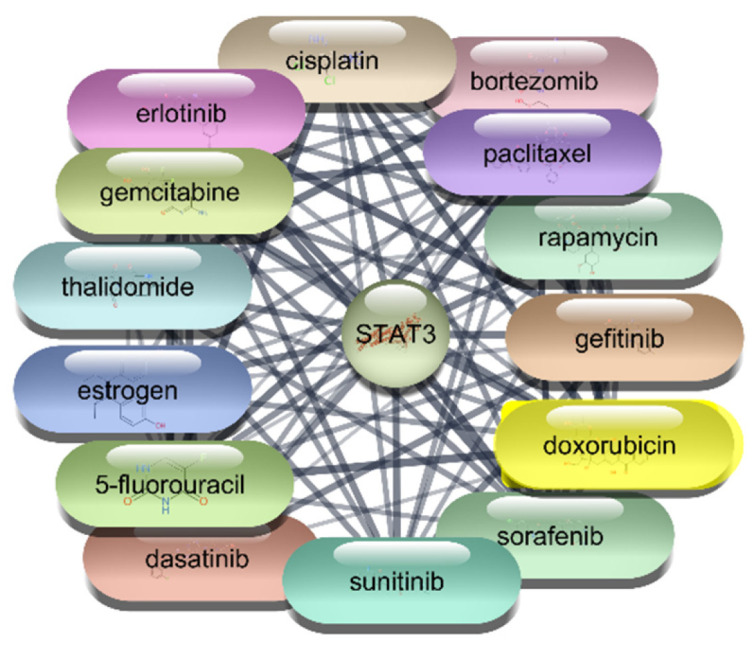
Top 15 FDA-Approved Drugs with Strongest Interactions with STAT-3.

Among the FDA-approved drugs screened for interactions with STAT3, doxorubicin emerged with the highest degree centrality of 23, underscoring its substantial connectivity within the network. Its high betweenness centrality of 55.19764 and closeness centrality of 0.4 highlight its significant role in facilitating interactions and efficient network reach. Paclitaxel, with a degree centrality of 21, shows strong connectivity, while its betweenness centrality of 26.38097 and closeness centrality of 0.393443 reflect its importance in the network’s interaction dynamics. Estrogen, with a degree centrality of 20 and the highest betweenness centrality of 74.17381, exhibits high connectivity and a pivotal role in the network, alongside a closeness centrality of 0.390244. Rapamycin, with a degree centrality of 18, significant betweenness centrality of 39.17713, and closeness centrality of 0.384, highlights its influence within the network. Similarly, 5-fluorouracil, cisplatin, and gefitinib, each with a degree centrality of 18 and varying betweenness and closeness centralities, demonstrate strong network presence and potential in targeting STAT3. Erlotinib and thalidomide, both with a degree centrality of 17, and respective betweenness and closeness centralities, reflect their roles in network dynamics and influence. Sorafenib, also with a degree centrality of 17, showcases its importance within the network with a betweenness centrality of 15.61034 and a closeness centrality of 0.380952. These metrics underscore the potential efficacy of these drugs as therapeutic agents targeting STAT3. The analysis reveals that drugs like doxorubicin, paclitaxel, and estrogen are among the most influential within the STAT3 interaction network. Their high centrality metrics suggest they hold considerable potential as therapeutic agents for targeting STAT3 in the context of RA which is shown in Table 4, Figure 6 and Figure 7. These top 10 drugs will undergo further molecular docking studies to validate their interactions and explore their therapeutic efficacy.

#### 3.2.4. TNF-α Protein–Drug Interaction Analysis

For the TNF-α protein, we conducted an extensive screening of 2637 FDA-approved drugs, identifying 105 drugs that demonstrated direct interactions with TNF-α. These interactions were further analyzed using Cytoscape software, which allowed us to construct and evaluate a comprehensive protein–drug interaction network. The analysis employed centrality metrics such as degree, betweenness, and closeness to assess the significance and impact of each drug within the network. Degree centrality reflects the number of direct connections a drug has within the network, highlighting its connectivity. Betweenness centrality measures a drug’s role in facilitating interactions between other nodes, indicating its influence in the network’s communication pathways. Closeness centrality indicates how efficiently a drug can interact with other nodes, showing its overall network accessibility. These metrics are pivotal for understanding the influence of each drug and its potential efficacy in targeting TNF-α. The detailed analysis of these metrics provides insight into the therapeutic potential of these drugs for targeting TNF-α, guiding further research and drug repurposing efforts.

**Figure 8:**
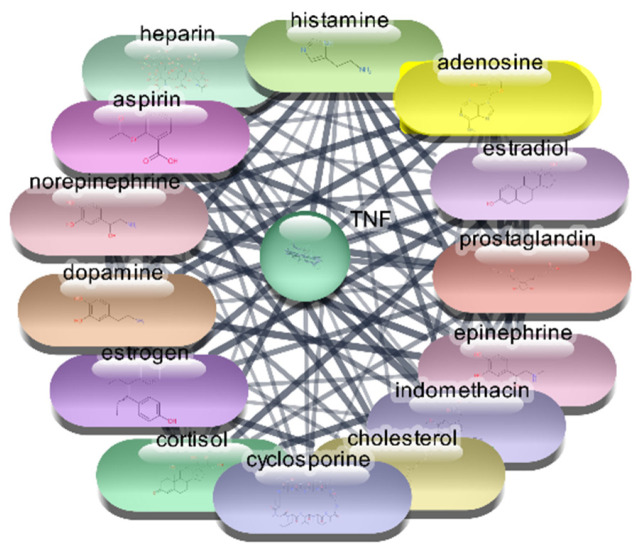
Top 15 FDA-Approved Drugs with Strongest Interactions with TNF-α.

The analysis of the top 10 FDA-approved drugs interacting with TNF-α reveals significant insights into their potential therapeutic efficacy. Cholesterol stands out with the highest degree centrality of 53, indicating the most direct interactions within the network. Its high betweenness centrality of 409.4760674 and closeness centrality of 0.666666667 underscore its critical role in network connectivity and efficiency. Aspirin, with a degree centrality of 45 and a notable betweenness centrality of 276.6920675, also shows strong connectivity and influence. Norepinephrine (degree 43), epinephrine (degree 41), and histamine (degree 40) demonstrate substantial network presence, reflected by their high betweenness and closeness centrality scores, suggesting their potential as effective TNF-α inhibitors. Estrogen, cyclosporine, prostaglandin, heparin, and dopamine, with degree centralities ranging from 39 to 37, further contribute to the network’s robustness, each playing a pivotal role in facilitating interactions and ensuring efficient communication within the network which is shown Table 5 and Figure 8. These centrality metrics collectively highlight the potential of these drugs in targeting TNF-α, providing a strong foundation for further research and drug repurposing efforts aimed at treating conditions mediated by TNF-α.

#### 3.2.5. Interleukin-6 (IL-6) Protein–Drug Interaction Analysis

For the IL-6 protein, we conducted a comprehensive screening of 2637 FDA-approved drugs, identifying 97 drugs that demonstrated direct interactions with the IL-6 protein. These interactions were further analyzed using Cytoscape software, which allowed us to construct and evaluate an extensive protein–drug interaction network. The analysis employed centrality metrics such as degree, betweenness, and closeness to assess the significance and impact of each drug within the network. Degree centrality highlights a drug’s connectivity by reflecting the number of direct connections it has within the network. Betweenness centrality measures a drug’s role in facilitating interactions between other nodes, indicating its influence in the network’s communication pathways. Closeness centrality indicates how efficiently a drug can interact with other nodes, showing its overall network accessibility. These metrics are crucial for understanding each drug’s influence and potential efficacy in targeting IL-6. The detailed analysis of these metrics provides valuable insights into the therapeutic potential of these drugs for targeting IL-6, guiding further research and drug repurposing efforts.

**Figure 9:**
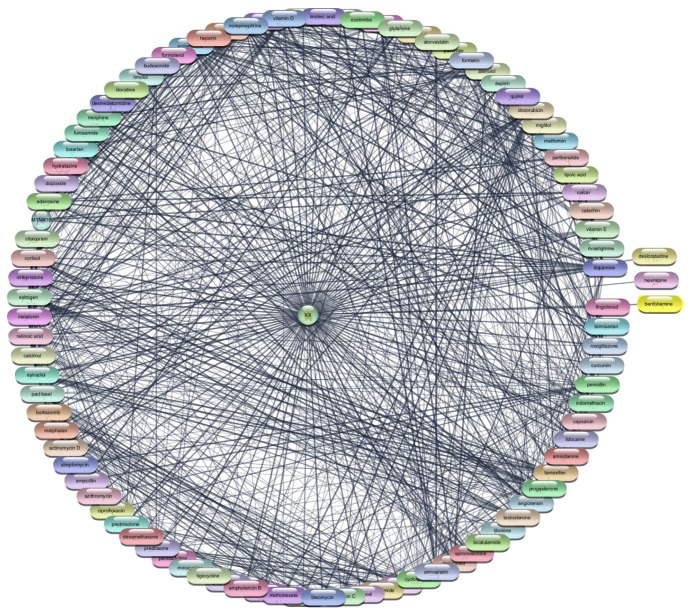
Interaction Network of 97 FDA-Approved Drugs with IL-6.

**Figure 10:**
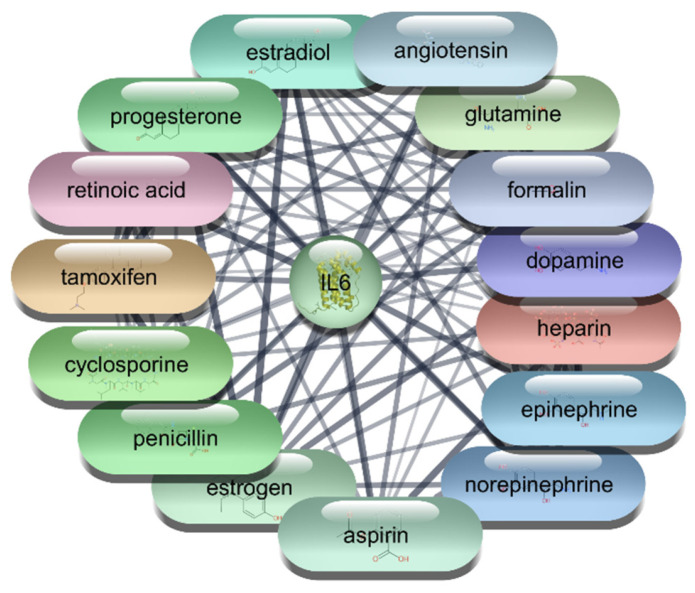
Top 15 FDA-Approved Drugs with Strongest Interactions with IL-6.

The analysis of the top 10 FDA-approved drugs interacting with IL-6 reveals several significant findings. Estrogen leads the list with a degree centrality of 37, indicating it has the highest number of direct connections within the network. This is followed closely by Aspirin and Formalin, each with a degree centrality of 36, showing their substantial connectivity. Cyclosporine, Norepinephrine, and Epinephrine also exhibit strong network presence with degree centrality values of 35 and 34, respectively. The high betweenness centrality of Norepinephrine (131.56) suggests it plays a crucial role in facilitating interactions between other nodes, highlighting its influence within the network. Heparin, with a betweenness centrality of 102.59, also demonstrates a significant impact on network communication pathways. Closeness centrality metrics, such as those for Estrogen (0.66) and Aspirin (0.63), indicate these drugs have efficient network accessibility, enabling quick interactions with other nodes. Retinoic acid, Progesterone, and Angiotensin, all with degree centrality of 33, further underscore their importance within the IL-6 interaction network which is shown in Table 6, Figure 9 and Figure 10. This comprehensive analysis underscores the therapeutic potential of these drugs in targeting IL-6, providing a strong foundation for future research and drug repurposing efforts to mitigate IL-6 mediated conditions.

### 3.3. Molecular Docking Studies

In the next phase of our research, we conducted molecular docking studies to further evaluate the interactions between the selected proteins (IL-1β, IL-6, TNF-α, AKT1, and STAT3) and their corresponding FDA-approved drugs identified from the protein–drug interaction analysis. Molecular docking is a crucial computational technique that predicts the preferred orientation of a drug molecule when bound to its target protein, providing insights into the binding affinity and interaction dynamics. By simulating these interactions, we aimed to identify the most promising drug candidates based on their binding scores and interaction profiles. This approach allows for a detailed understanding of the molecular mechanisms underlying drug efficacy and aids in the optimization of therapeutic strategies for targeting these key proteins involved in inflammatory and autoimmune pathways. The docking studies not only validate the initial network analysis but also provide a robust platform for selecting potential drugs for further experimental validation and clinical applications.

#### 3.3.1. Selection of Target Proteins for Molecular Docking Studies

For our comprehensive study focused on RA, we selected five key proteins IL-1β, IL-6, TNF-α, AKT1, and STAT3 based on a meticulous network pharmacology drug repurposing analysis. These proteins were identified as critical nodes in the PPI network, underscoring their central roles in the pathogenesis of RA, particularly in inflammatory and autoimmune pathways. To further elucidate their therapeutic potential, we retrieved their structural data from the PDB and utilized the PLIP web server to pinpoint their precise binding sites. For IL-1β (PDB ID: 1ITB), key binding residues include Arg11, Ser13, Gln15, Met20, Gly22, Lys27, Leu29, Met36, Gln38, Gln126, Pro131, Thr147, and Gln149. IL-6 (PDB ID: 1ALU) features binding sites such as Arg40, Glu51, Gln75, Ser76, Gln156, His164, Arg168, Gln175, Arg179, Arg182, and Gln183. TNF-α (PDB ID: 2AZ5) binds significantly at residues Tyr59, Tyr119, and Tyr151. STAT3 (PDB ID: 6NJS) shows crucial interactions at Arg609, Glu612, Ser613, Glu638, Pro639, Tyr640, Gln644, and Leu666, while AKT1 (PDB ID: 3O96) binds at Asn54, Trp80, Ile84, Ser205, Leu210, Leu264, Lys268, Tyr272, and Asp292. These specific binding sites, identified through PLIP, provide a robust foundation for our molecular docking studies, allowing us to assess the binding affinities and interaction profiles of FDA-approved drugs with these critical proteins. This targeted approach aims to optimize therapeutic strategies, offering promising avenues for repurposing existing drugs to effectively modulate these pivotal proteins and address the underlying mechanisms of RA.

#### 3.3.2. Screening and Selection of Ligands for Molecular Docking

We conducted an extensive screening of 2637 FDA-approved drugs to identify potential therapeutic agents that directly interact with key proteins involved in RA. For the IL-1β protein, we identified 65 drugs with direct interactions. Similarly, for AKT1, our screening revealed 106 drugs, while for STAT3, we identified 49 drugs. The TNF-α protein showed direct interactions with 105 drugs, and the IL-6 protein had 97 drugs demonstrating direct interactions. All these drugs, which showed direct interactions with their respective target proteins, were selected for molecular docking studies. This process is designed to enhance our understanding of how these drugs interact at a molecular level, potentially leading to more effective therapeutic strategies. By utilizing molecular docking, we aim to elucidate the binding affinities and specific interactions between these FDA-approved drugs and their target proteins, thereby identifying promising candidates for repurposing in the treatment of RA.

#### 3.3.3. Interpretation of Molecular Docking Results of TNF-α

For the TNF-α protein (PDB ID: 2AZ5), molecular docking studies revealed several FDA-approved drugs with significant binding affinities. Rifampicin exhibited the highest binding affinity at −9.8 kcal/mol, suggesting strong interactions with TNF-α, potentially repurposing its antimicrobial effects to modulate inflammatory responses. Azelastine, typically an antihistamine, showed a notable binding affinity of −7.2 kcal/mol, indicating potential anti-inflammatory applications. Testosterone, with a binding affinity of −6.9 kcal/mol, may offer insights into gender-specific therapeutic effects on inflammation. Berberine and Troglitazone, both with a binding affinity of −6.6 kcal/mol, indicate potential repurposing opportunities for this antimicrobial and antidiabetic agent, respectively. The co-crystal standard exhibited a binding affinity of −5.9 kcal/mol, serving as a baseline for comparison.

#### 3.3.4. IL-6

In the case of the IL-6 protein (PDB ID: 1ALU), docking results identified several promising drug candidates. Telmisartan, an antihypertensive agent, demonstrated the highest binding affinity at −8.1 kcal/mol, suggesting its potential to inhibit IL-6 mediated inflammatory pathways. Tibolone, a synthetic steroid, showed a binding affinity of −7.5 kcal/mol, indicating possible uses in modulating immune responses in addition to its hormonal replacement therapy role. Actinomycin and Doxorubicin, both with binding affinities of −7.4 kcal/mol, are traditionally used as chemotherapeutic agents, hinting at their potential repurposing for anti-inflammatory effects. Cortisol, with a binding affinity of −6.3 kcal/mol, reinforces its known anti-inflammatory properties. The co-crystal standard had the lowest binding affinity of −4.7 kcal/mol.

#### 3.3.5. IL-1β

For IL-1β (PDB ID: 1ITB), docking studies identified Danazol with a binding affinity of −7.9 kcal/mol, highlighting its potential repurposing from treating endometriosis to modulating inflammatory responses. Troglitazone, an antidiabetic drug, showed a binding affinity of −7.5 kcal/mol, suggesting its efficacy in inhibiting IL-1β mediated pathways. Glibenclamide, traditionally used for diabetes, demonstrated a binding affinity of −6.1 kcal/mol, while Tamoxifen and Tacrolimus, with binding affinities of −4.4 kcal/mol each, indicated potential repurposing from their original uses in breast cancer treatment and immunosuppression, respectively. The co-crystal standard had a binding affinity of −4.3 kcal/mol.

#### 3.3.6. AKT1

The AKT1 protein (PDB ID: 3O96) showed exceptionally strong interactions with several drugs. The co-crystal ligand exhibited the highest binding affinity at −14.9 kcal/mol, setting a high benchmark. Etoposide, with a binding affinity of −13 kcal/mol, and Ponatinib, at −12.6 kcal/mol, both chemotherapeutic agents, suggest significant potential in targeting AKT1. Imatinib and Novobiocin, with binding affinities of −11.5 kcal/mol each, indicate potential repurposing for kinase inhibition and antimicrobial activity, respectively. Daunorubicin, with a binding affinity of −9 kcal/mol, traditionally used in chemotherapy, further underscores the potential for repurposing in targeting AKT1 pathways.

#### 3.3.7. STAT3

For STAT3 (PDB ID: 6NJS), docking results highlighted Pimozide with a binding affinity of −7.5 kcal/mol, indicating its potential repurposing from antipsychotic use to STAT3 inhibition. Alectinib and Rapamycin, both with binding affinities of −7.1 kcal/mol, suggest applications in targeting STAT3 mediated pathways, expanding their roles beyond cancer treatment and immunosuppression. Sorafenib, with a binding affinity of −6.9 kcal/mol, and Dasatinib, at −6.7 kcal/mol, further reinforce the potential for repurposing these kinase inhibitors for STAT3 targeting. The co-crystal standard also had a binding affinity of −6.7 kcal/mol, providing a comparative baseline which are shown in Table 7.

### 3.4. Prioritizing FDA-Approved Drugs for RA Treatment

In conclusion, Rifampicin, traditionally an anti-tubercular drug, emerges as a promising candidate for repurposing to target TNF-α in RA therapy, due to its strong binding affinity of −9.8 kcal/mol. Telmisartan, commonly used as an antihypertensive, shows a significant binding affinity of −8.1 kcal/mol, suggesting its potential repurposing for targeting IL-6 in RA. Danazol, typically used for endometriosis and fibrocystic breast disease, exhibits a binding affinity of −7.9 kcal/mol, making it a viable candidate for repurposing to inhibit IL-1β in RA treatment. Pimozide, an antipsychotic drug, demonstrates a notable binding affinity of −7.5 kcal/mol, highlighting its potential to be repurposed for targeting STAT3 in RA. Finally, Etoposide, a chemotherapeutic agent, shows a strong binding affinity of −13 kcal/mol, suggesting its possible repurposing for inhibiting AKT1 in RA therapy. These findings underscore the potential efficacy of these FDA-approved drugs when repurposed for RA, based on their robust interactions with the respective protein targets.

### 3.5. 2D Interactions

**Figure 11:**
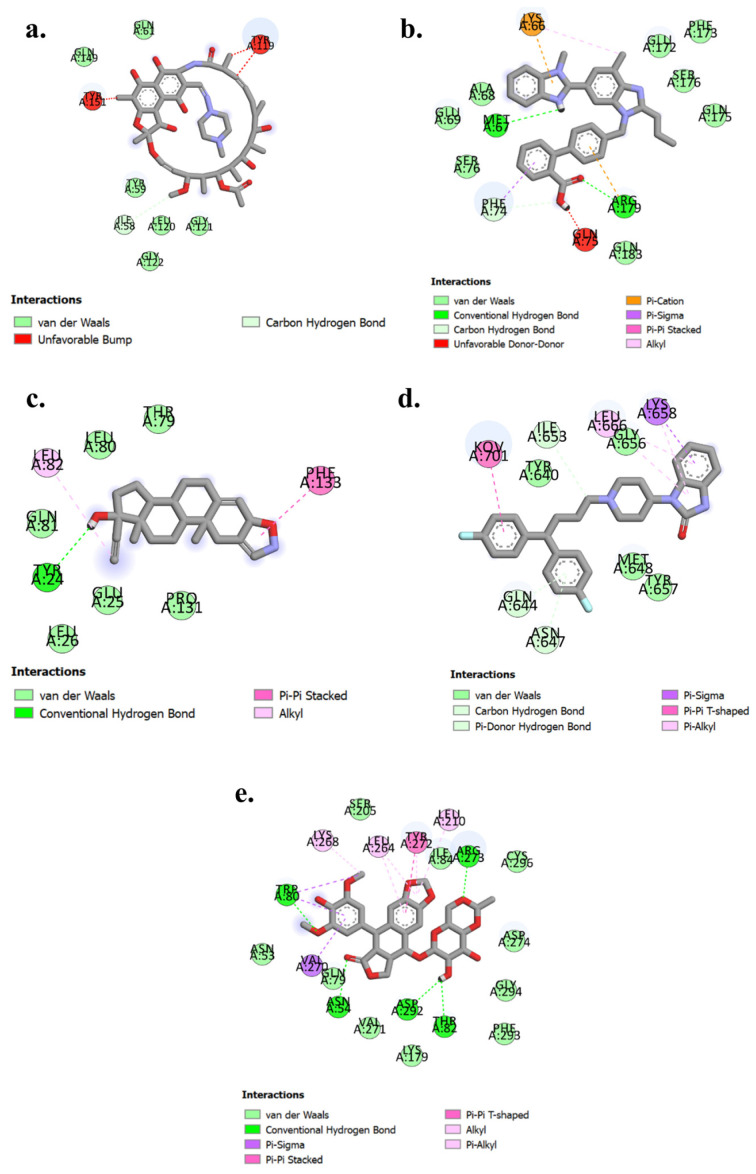
2D interaction diagrams of (**a**) 2AZ5- Rifampicin (**b**) 1ALU-Telmesartan (**c**) 1ITB-Danazol. (**d**) 6NJS-Pimazide (**e**) 3O96-Etoposide.

For TNF-α (PDB ID: 2AZ5), Rifampicin exhibits notable interactions, including Van der Waals forces with residues Gln61, Gln149, Tyr59, Leu120, Gly121, and Gly122, indicating a stable binding environment. Additionally, it forms carbon-hydrogen bond interactions with Ile58, further stabilizing the complex. However, unfavorable interactions with Tyr119 and Tyr151 suggest areas for potential optimization in enhancing drug efficacy which is shown in Figure 11a. For IL-6 (PDB ID: 1ALU), Telmisartan demonstrates conventional hydrogen bonding with Met67 and Arg179, contributing to a strong binding affinity. It also engages in Van der Waals interactions with Ala68, Glu69, Ser76, Glu172, Phe173, Ser176, Gln175, and Gln183, enhancing stability within the binding site. Additionally, carbon-hydrogen bonding with Phe74 and a pi-cation interaction with Lys66 further stabilize the complex, despite the unfavorable interaction with Gln75 which is shown in Figure 11b. For IL-1β (PDB ID: 1ITB), Danazol forms conventional hydrogen bonds with Tyr24. It engages in Van der Waals interactions with Glu25, Leu26, Pro131, Gln81, Leu80, and Thr79. Additionally, Danazol stabilizes its binding through allyl interactions with Leu82 and pi-pi stacked interactions with Phe133 which is shown in Figure 11c. For STAT3 (PDB ID: 6NJS), Pimozide demonstrates binding through Van der Waals interactions with Tyr640, Gly656, Met648, and Tyr657. It forms carbon-hydrogen bonds with Gln644, Asn647, and Ile653. Additionally, Pimozide interacts with Leu666 via pi-alkyl interactions and Lys658 through pi-sigma interactions which is shown in Figure 11d. For AKT1 (PDB ID: 3O96), Etoposide binds effectively with Arg272, Trp80, Asn54, Asp292, and Thr82 through conventional hydrogen bonds. It also exhibits Van der Waals interactions with Ser205, Ile84, Cys296, Asn53, Gln79, Val271, Lys179, Phe293, Gly294, and Asp274. Alkyl/pi-alkyl interactions are observed with Lys268, Leu264, and Leu210. Additionally, Tyr272 participates in pi-pi-stacked interactions, while Val270 forms pi-sigma interactions which is shown in Figure 11e.

### 3.6. MD Simulations for Top Compounds for Each Target

MD simulation studies were conducted to analyze the stability and dynamic behavior of ligand-protein complexes under a simulated biological environment. These simulations provide insights into the kinetics of ligand binding within the enzyme’s active site and help assess the conformational stability of the complexes. The study involved five target proteins implicated in RA, each paired with a repurposed drug candidate selected based on docking scores. The final ligand-protein complexes included: TNF-α -Rifampicin, IL-6-Telmisartan, IL-1β-Danazol, AKT-1-Etoposide, and STAT-3-Pimozide. Each complex underwent a 100 ns MD simulation for detailed evaluation.

#### 3.6.1. Root Mean Square Deviation (RMSD)

**Figure 12:**
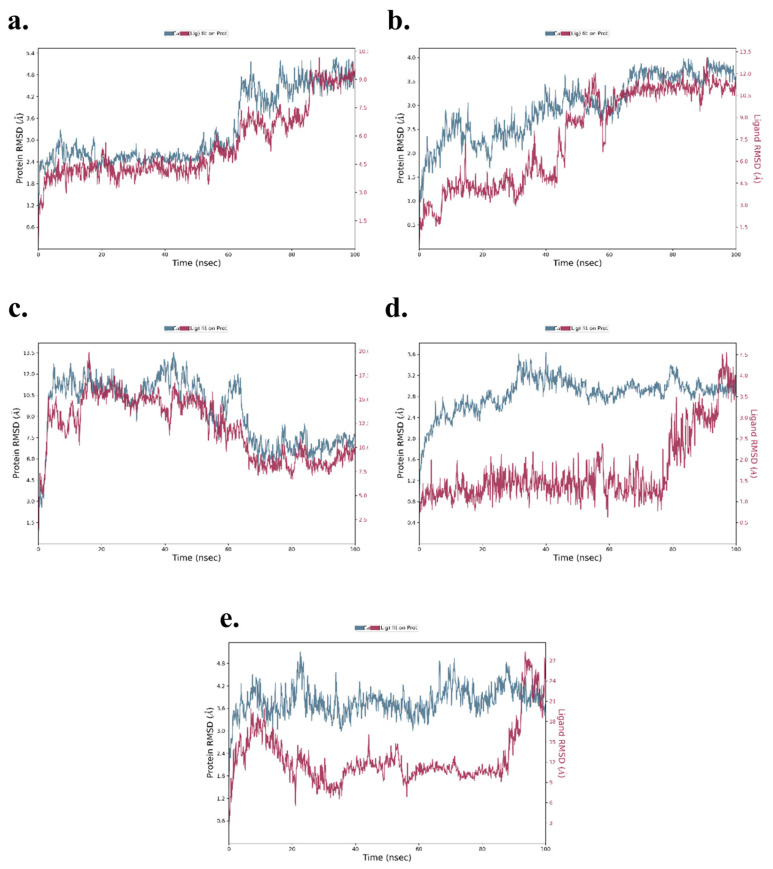
RMSD of (**a**) TNF-α -Rifampicin Complex (**b**) IL-6-Telmisartan Complex (**c**) IL-1β-Danazol Complex (**d**) AKT-1-Etoposide complex (**e**) STAT-3-Pimozide Complex.

RMSD is a critical parameter in MD simulations, measuring the structural deviation of protein backbone atoms (Cα) and the ligand over time. It provides a comprehensive understanding of the complex’s stability and flexibility during the simulation. The RMSD analysis for each complex is as follows: TNF-α -Rifampicin Complex—The RMSD of Cα atoms exhibited initial fluctuations in the range of 2.2–3.0 Å during the first 60 ns, stabilizing slightly in the range of 3.8–4.8 Å for the next 40 ns. Meanwhile, the RMSD for the ligand fit to the protein fluctuated between 3.5–4.8 Å during the initial 60 ns and increased to a range of 6–9 Å over the next 40 ns, where it eventually stabilized. These findings indicate a stable ligand-protein interaction with moderate fluctuations. The data is depicted in Figure 12a. IL-6-Telmisartan Complex—The Cα atoms exhibited medium fluctuations during the first 60 ns, stabilizing within a range of 3.2–3.8 Å over the subsequent 40 ns. However, the ligand RMSD showed high fluctuations in the initial 60 ns, eventually stabilizing with minor deviations in the range of 10.5–12.0 Å during the final 40 ns. This indicates a steady binding mode after initial dynamic adjustments, as shown in Figure 12b. IL-1β-Danazol Complex—For this complex, the Cα atoms displayed high fluctuations during the initial 70 ns, stabilizing within 6.5–9.0 Å in the final 30 ns. Similarly, the ligand RMSD exhibited significant fluctuations for the first 70 ns, with stabilization occurring in the range of 7.5–10.0 Å during the last 30 ns. The overall results highlight a delayed stabilization process, illustrated in Figure 12c. AKT-1-Etoposide Complex—This complex showed persistent high fluctuations in the Cα atoms throughout the 100 ns simulation, with only brief periods of stability. In contrast, the ligand RMSD demonstrated low fluctuations in the range of 1.0–2.0 Å during the first 80 ns, stabilizing at 2.0–4.0 Å in the final 20 ns. This suggests that while the protein exhibited significant dynamics, the ligand maintained relatively stable binding, as depicted in Figure 12d. STAT-3-Pimozide Complex—The Cα atoms exhibited minimal fluctuations throughout the 100 ns, maintaining a range of 3.6–4.8 Å. However, the ligand RMSD displayed extreme fluctuations ranging from 3–27 Å, indicating a highly unstable ligand binding. This result reflects poor binding stability for the complex, as shown in Figure 12e. The RMSD analysis of the MD simulations provides valuable insights into the stability of the ligand-protein complexes. Among the evaluated complexes, the TNF-α -Rifampicin complex demonstrated superior stability, with moderate initial fluctuations that stabilized effectively over time, suggesting a robust binding interaction. In contrast, the AKT-1-Etoposide complex exhibited persistent fluctuations throughout the simulation, indicating a comparatively lower stability. The IL-6-Telmisartan and IL-1β-Danazol complexes showed significant initial fluctuations but eventually stabilized, reflecting moderate binding interactions. However, the STAT-3-Pimozide complex displayed substantial instability throughout the simulation, with large RMSDs, indicating weak binding interactions. Overall, the TNF-α -Rifampicin complex emerges as the most stable, followed by IL-6-Telmisartan and IL-1β-Danazol, with AKT-1-Etoposide and STAT-3-Pimozide showing lower stability.

#### 3.6.2. Root Mean Square Fluctuation (RMSF)

**Figure 13:**
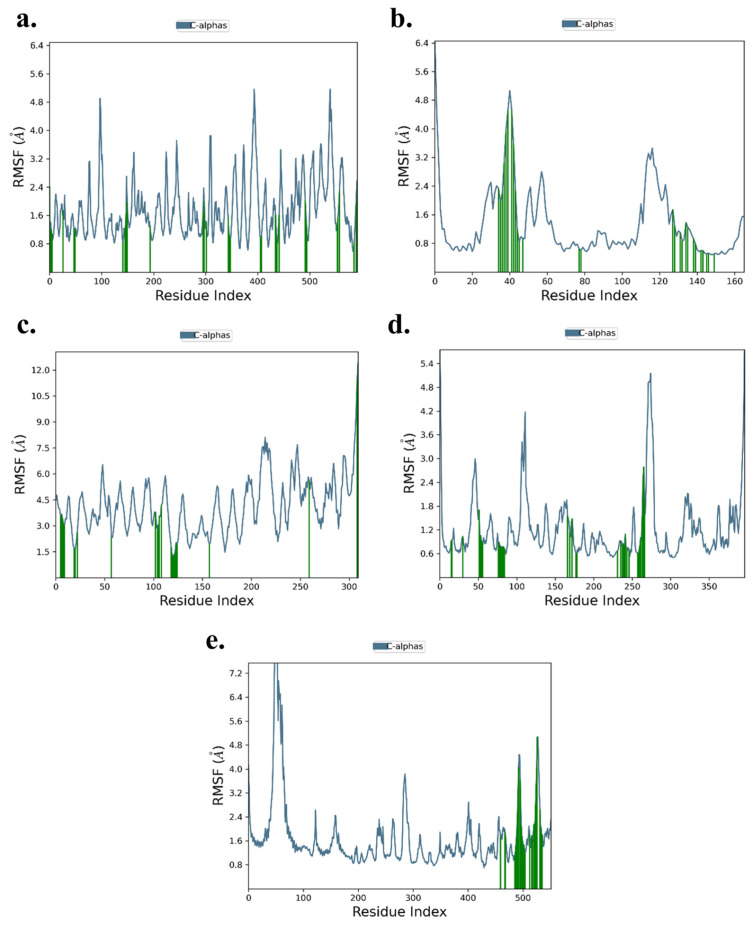
RMSF of (**a**) TNF-α -Rifampicin Complex (**b**) IL-6-Telmisartan Complex (**c**) IL-1β-Danazol Complex (**d**) AKT-1-Etoposide complex (**e**) STAT-3-Pimozide Complex.

The RMSF analysis highlights local changes in amino acid residues along the protein chains for the five ligand-protein complexes. For the TNF-α -Rifampicin complex, fluctuations ranged between 1.0–4.8 Å, while for the IL-6-Telmisartan complex, the range was slightly lower at 0.8–4.8 Å. The IL-1β-Danazol complex exhibited relatively higher fluctuations, ranging from 1.5–6.0 Å. Both the AKT-1-Etoposide and STAT-3-Pimozide complexes displayed similar fluctuation ranges of 0.6–4.8 Å and 0.8–4.8 Å, respectively. Importantly, across all five complexes, minimal fluctuations were observed at the binding sites, suggesting stable interactions between the ligands and their respective target proteins. This stability at the binding sites reinforces the reliability of the docking results, as shown in Figure 13a–e.

#### 3.6.3. Protein–Ligand Contacts Analysis

**Figure 14:**
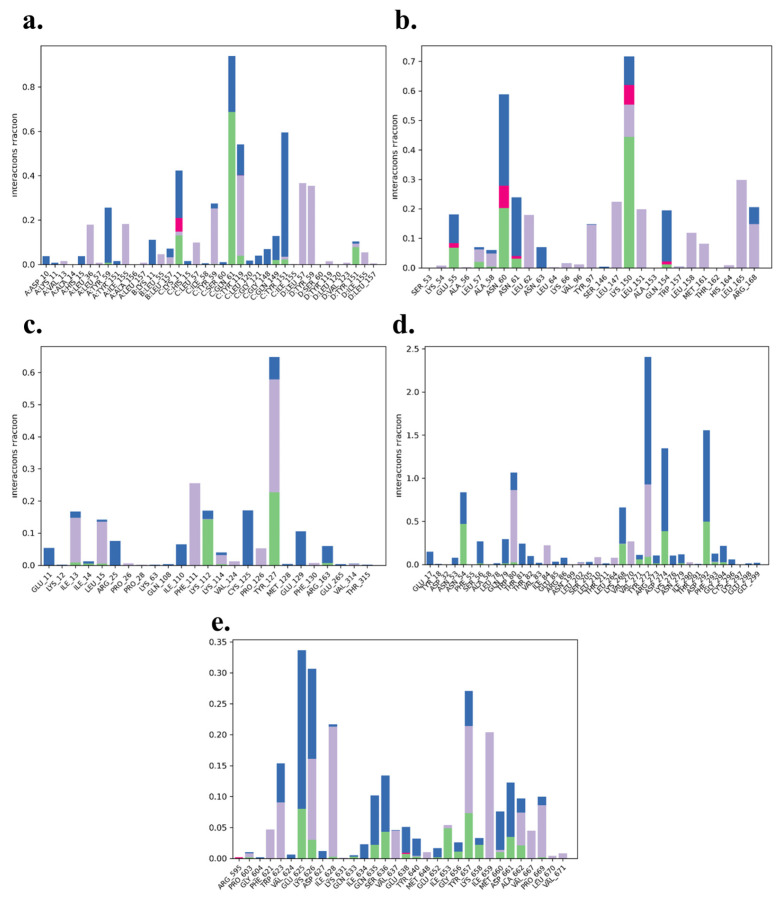
Protein ligand contacts of (**a**) TNF-α -Rifampicin Complex (**b**) IL-6-Telmisartan Complex (**c**) IL-1β-Danazol Complex (**d**) AKT-1-Etoposide complex (**e**) STAT-3-Pimozide Complex.

The analysis of protein–ligand contacts revealed distinct interaction patterns across the five complexes, with hydrophobic interactions dominating but intermixed with key hydrogen bonding and water bridge interactions that contribute to the stability and specificity of the binding. The TNF-α -Rifampicin complex exhibited a strong network of hydrophobic interactions, with residues such as Lys11, Gln61, Tyr119, Gln149, and Tyr151 contributing moderate to significant hydrogen bonding. The normalized stacked bar chart indicated that 80% of the simulation time maintained these interactions, underscoring the complex’s robustness and ligand affinity throughout the trajectory. This combination of stable hydrophobic and hydrogen bonding interactions reflects a well-balanced binding profile for Rifampicin with TNF-α. The IL-6-Telmisartan complex was characterized by a blend of hydrophobic interactions alongside notable hydrogen bonding contributions from Glu55, Leu57, Asn60, Asn61, and Lys150. Lys150, in particular, exhibited a strong hydrogen bond interaction fraction, while water bridge interactions with residues such as Asn63 and Arg168 further enhanced the binding stability. Despite moderate fluctuations, the interactions persisted for 70% of the simulation time, indicating a reliable yet dynamic binding relationship between Telmisartan and IL-6. The IL-1β-Danazol complex demonstrated a mix of hydrophobic and moderate hydrogen bonding interactions. Key residues involved included Lys12, Leu15, Lys112, Tyr127, Arg163, and Glu129, which formed transient but notable interactions, complemented by water bridges. This complex-maintained interactions for 60% of the simulation time, suggesting moderate binding stability. The prevalence of water-mediated interactions highlights a dynamic and adaptable binding environment for Danazol with IL-1β. In the AKT-1-Etoposide complex, hydrophobic interactions with residues such as Trp80, Ile84, Leu210, Leu264, Val270, Tyr272, and Ile290 were predominant. Moderate hydrogen bonding interactions were also observed with Asn54, Trp80, Lys268, Val271, Asp274, and Asp292, though they were maintained for only a short duration. The overall interaction stability was weaker, with just 25% of the simulation time preserving these contacts. This highlights a more transient and less stable binding nature for Etoposide with AKT-1. The STAT-3-Pimozide complex displayed minimal hydrophobic and hydrogen bonding interactions, with residues maintaining these contacts for only 35% of the simulation time. This limited interaction persistence underscores a relatively unstable binding affinity for Pimozide with STAT-3, suggesting room for optimization in ligand design to enhance stability and efficacy. Overall, the TNF-α -Rifampicin complex demonstrated the most stable and persistent interaction profile, maintaining contacts for 80% of the simulation time, followed by the IL-6-Telmisartan complex at 70%. The IL-1β-Danazol complex exhibited moderate stability at 60%, whereas the AKT-1-Etoposide and STAT-3-Pimozide complexes showed weaker interaction persistence, with 25% and 35% of the simulation time maintaining contacts, respectively. These findings provide valuable insights into the binding dynamics and potential optimization strategies for the evaluated ligand-protein complexes. All the protein ligand complexes are shown in Figure 14a–e.

The MD simulations, encompassing RMSD, RMSF, and protein–ligand contact analyses, revealed that the TNF-α -Rifampicin and IL-6-Telmisartan complexes exhibited superior stability with consistent interactions and minimal binding site fluctuations. The IL-1β-Danazol complex showed moderate stability, with slightly higher fluctuations yet retaining key interactions over the trajectory. Conversely, AKT-1-Etoposide and STAT-3-Pimozide complexes demonstrated weaker interaction consistency and higher fluctuations, indicating comparatively lower stability. These findings offer a comprehensive understanding of the dynamic behavior of the studied complexes, highlighting TNF-α and IL-6 as promising targets for further exploration.

## 4. Discussion

The high cost and extended timeline of de novo drug development necessitate innovative strategies for discovering new therapies for complex diseases like Rheumatoid Arthritis (RA). In this study, we implemented an integrated computational drug repurposing pipeline, combining network pharmacology, molecular docking, and molecular dynamics (MD) simulations to systematically evaluate 2637 FDA-approved drugs for their potential to target key proteins in RA pathogenesis. Our workflow began with the construction of a Protein–Protein Interaction (PPI) network from RA-associated genes, which robustly identified TNF-α, IL-6, IL-1β, STAT3, and AKT1 as topologically central hubs. This finding aligns perfectly with the established understanding of RA, where these proteins are recognized as master regulators of inflammation, immune cell activation, and synovial hyperplasia [1,2,12]. The subsequent Protein–drug Interaction (PDI) network analysis served as an unbiased screening tool, capturing a wide spectrum of compounds based on their interaction potential with these targets. A particularly insightful outcome of this unbiased screen was the high ranking of the chemotherapeutic taxanes, paclitaxel and docetaxel, as top interactors for the AKT1 node. While the conventional use of these cytotoxic agents at high doses is inappropriate for chronic diseases, our computational finding is strongly corroborated by a compelling body of recent pre-clinical evidence. Notably, studies from the last five years have demonstrated that paclitaxel significantly inhibits the migration of rheumatoid arthritis fibroblast-like synoviocytes (RA-FLS) and reduces the production of critical inflammatory mediators, including IL-6, IL-8, and IL-1β [49]. Furthermore, in rodent models of collagen-induced arthritis (CIA), paclitaxel has been shown to alleviate synovitis and bone destruction, with its mechanism linked to the direct inhibition of the AKT/mTOR pathway [49,50]. Similarly, docetaxel has recently been reported to reduce disease progression and joint destruction in rat polyarthritis models, an effect associated with the suppression of key inflammatory cytokines like TNF-α and IL-1β [51,52]. The convergence of our unbiased computational results with these independent experimental findings provides a powerful validation of our network pharmacology approach and highlights the potential for low-dose, immunomodulatory applications of these agents. Following this initial network-based discovery, we employed molecular docking and 100 ns MD simulations to rigorously evaluate the binding affinity and stability of the top candidate drugs. This step served as a critical filter to move from network-predicted interactions to mechanistically plausible binding modes. Our results identified Rifampicin, Telmisartan, Danazol, and Pimozide as the most promising repurposing candidates, demonstrating strong binding affinities and stable dynamics with TNF-α, IL-6, IL-1β, and STAT3, respectively. The MD simulations provided a deeper, dynamic validation beyond static docking. The TNF-α-Rifampicin and IL-6-Telmisartan complexes exhibited superior stability, with low RMSD values and persistent protein–ligand contacts throughout the simulation trajectory. This suggests a robust and specific interaction, reinforcing their potential as effective inhibitors. In contrast, the STAT-3-Pimozide complex showed higher flexibility, indicating a more dynamic binding mode that may inform future optimization. Finally, our multi-layered computational strategy successfully bridged the gap between large-scale network prediction and atomistic interaction analysis. The workflow not only identified clinically tractable candidates like Rifampicin and Telmisartan but also demonstrated its predictive power by independently highlighting the emerging role of taxanes in RA, as supported by the latest pre-clinical research. A critical advantage of our final selected candidates is their established clinical profile. For instance, Rifampicin has a long history of use in long-term tuberculosis prophylaxis, and Telmisartan is a widely used antihypertensive with a well-tolerated safety profile. This balance between predicted efficacy (strong binding affinity and complex stability) and inherent clinical translatability (known pharmacokinetics and manageable safety profiles for chronic use) is the core value proposition of our repurposing strategy. While the significant clinical hurdles of dose-finding and long-term safety profile the taxanes for RA treatment, they represent a highly justified, mechanistically grounded avenue for future investigation. Therefore, the final candidates, especially Rifampicin and Telmisartan, represent high-priority targets for future experimental work, where their predicted anti-arthritic activity can be tested in relevant biological models.

## 5. Conclusions

In conclusion, our comprehensive study underscores the potential of drug repurposing as an innovative strategy for identifying novel therapeutic agents for RA. By repurposing existing FDA-approved drugs, we can accelerate the drug discovery process while minimizing the risks and costs associated with developing new compounds. Through PPI and protein–drug interaction analyses, followed by molecular docking studies, we identified Rifampicin, Telmisartan, Danazol, Pimozide, and Etoposide as promising candidates for repurposing. These drugs demonstrated strong binding affinities to TNF-α, IL-6, IL-1β, STAT3, and AKT1, suggesting their potential to modulate key inflammatory pathways involved in RA. Additionally, the results from MD simulations provided valuable insights into the stability and interaction dynamics of the protein–ligand complexes. Rifampicin and Telmisartan showed exceptional stability and minimal fluctuations, reinforcing their potential as effective candidates for targeting TNF-α and IL-6 in RA therapy. While other complexes demonstrated moderate stability, they still exhibited promising binding interactions, indicating the potential for further optimization. This integrated approach of combining computational techniques with experimental validation not only offers a cost-effective and time-efficient solution but also paves the way for the development of more effective RA treatments. By incorporating both molecular docking and MD studies, we further emphasize the importance of computational and experimental methods in advancing drug discovery for RA, offering a new frontier for therapeutic interventions.

### Future Aspects

The findings from this integrated computational study provide a strong rationale for the experimental validation of the identified drug candidates. The primary candidates emerging from this study, particularly Rifampicin and Telmisartan, present a compelling and lower-risk proposition for immediate experimental validation due to their favorable safety profiles and robust computational scores. As a direct and immediate follow-up to this work, we plan to prioritize these two candidates for in vitro biological evaluation. This will involve testing their ability to inhibit the production and activity of their respective targets (TNF-α and IL-6) in relevant cellular models of RA, such as cytokine-stimulated human fibroblast-like synoviocytes (HFLS) or peripheral blood mononuclear cells (PBMCs). Subsequent in vivo studies in rodent models of Freund’s adjuvant-induced arthritis would be the logical next step to confirm therapeutic efficacy. This proposed validation pipeline underscores the practical value of our computational repurposing strategy in generating testable hypotheses for new RA treatments.

## Figures and Tables

**Table 1:** Top 20 Hub proteins in the PPI network based on degree, betweenness, and closeness centrality.

S. No	Gene	Degree	Betweenness	Closeness
1	TNF-α	913	167,860.9	0.033736
2	IL6	878	118,213.4	0.033715
3	IL1B	802	95,869.02	0.033672
4	AKT1	802	154,697.6	0.033683
5	GAPDH	770	169,260.9	0.033674
6	TP53	755	203,387.8	0.033664
7	ACTB	694	110,294.1	0.033631
8	IFNG	685	54,290.6	0.033608
9	CD4	685	71,873.13	0.033609
10	STAT3	679	74,866.05	0.033613
11	EGFR	665	128,590.9	0.033612
12	IL10	632	40,682.92	0.033578
13	ALB	619	80,364.31	0.033584
14	SRC	614	113,404	0.033579
15	MYC	595	82,344.4	0.03358
16	INS	581	79,854.29	0.033576
17	CTNNB1	576	99,586.8	0.033564
18	CD8A	567	36,863	0.033536
19	TLR4	559	38,275.69	0.033541
20	CXCL8	550	37,824.43	0.033529

**Table 2:** Top 10 FDA-Approved Drugs Interacting with IL-1β.

S. No	Gene	Degree	Betweenness	Closeness
1.	cyclosporine	26	0.614679	189.0437
2.	prostaglandin	22	0.59292	99.57395
3.	norepinephrine	22	0.59292	108.877
4.	indomethacin	21	0.59292	99.51681
5.	progesterone	21	0.59292	66.37792
6.	tetracycline	18	0.57265	204.2238
7.	cortisol	18	0.57265	60.78506
8.	estradiol	18	0.57265	166.0802
9.	heparin	18	0.57265	70.16999
10.	propranolol	14	0.54918	27.14841

**Table 3:** Top 10 FDA-Approved Drugs Interacting with AKT-1.

S. No	Gene	Degree	Betweenness	Closeness
1.	paclitaxel	36	112.8922	0.596591
2.	cisplatin	35	186.4022	0.59322
3.	doxorubicin	35	112.2757	0.59322
4.	imatinib	34	113.9072	0.589888
5.	lysine	33	248.9429	0.586592
6.	docetaxel	32	136.1355	0.58011
7.	rapamycin	30	102.9351	0.576923
8.	glutamine	30	123.1682	0.576923
9.	retinoic acid	29	86.78217	0.57377
10.	5-fluorouracil	28	53.25876	0.570652

**Table 4:** Top 10 FDA-Approved Drugs Interacting with STAT-3.

S. No	Gene	Degree	Betweenness	Closeness
1.	doxorubicin	23	55.19764	0.4
2.	paclitaxel	21	26.38097	0.393443
3.	estrogen	20	74.17381	0.390244
4.	rapamycin	18	39.17713	0.384
5.	5-fluorouracil	18	19.71496	0.384
6.	cisplatin	18	12.19764	0.384
7.	gefitinib	18	15.54209	0.384
8.	erlotinib	17	8.323832	0.380952
9.	thalidomide	17	15.80226	0.380952
10.	sorafenib	17	15.61034	0.380952

**Table 5:** Top 10 FDA-Approved Drugs Interacting with TNF-α.

S. No	Gene	Degree	Betweenness	Closeness
1.	cholesterol	53	409.4760674	0.666666667
2.	aspirin	45	276.6920675	0.634146341
3.	norepinephrine	43	206.2770861	0.626506024
4.	epinephrine	41	171.9433198	0.619047619
5.	histamine	40	217.0343707	0.615384615
6.	estrogen	39	135.9058147	0.611764706
7.	cyclosporine	38	162.5574692	0.608187135
8.	prostaglandin	38	150.5055708	0.611764706
9.	heparin	37	135.6237265	0.604651163
10.	dopamine	37	147.6741177	0.604651163

**Table 6:** Top 10 FDA-Approved Drugs Interacting with IL-6.

S. No	Gene	Degree	Betweenness	Closeness
1.	Estrogen	37	70.47	0.66
2.	Aspirin	36	88.01	0.63
3.	Formalin	36	75.62	0.62
4.	Cyclosporine	35	85.51	0.61
5.	Norepinephrine	34	131.56	0.61
6.	Epinephrine	34	87.99	0.61
7.	Retinoic acid	33	85.45	0.60
8.	Heparin	33	102.59	0.61
9.	Progesterone	33	85.60	0.60
10.	Angiotensin	33	42.86	0.60

**Table 7:** Molecular docking results of top 5 compounds for top 5 proteins.

S. No	Proteins	PDB ID	Ligands	Binding Affinities (kcal/mol)
1	TNF-α	2AZ5	Rifampicin	−9.8
			Azelastine	−7.2
			Testosterone	−6.9
			Berberine	−6.6
			Troglitazone	−6.6
			Co-Crystal	−5.9
2	IL6	1ALU	Telmisartan	−8.1
			Tibolone	−7.5
			Actinomycin	−7.4
			Doxorubicin	−7.4
			Cortisol	−6.3
			Co-Crystal	−4.7
3	IL-1β	1ITB	Danazol	-7.9
			Troglitazone	−7.5
			Glibenclamide	−6.1
			Tamoxifen	−4.4
			Tacrolimus	−4.4
			Co-Crystal	−4.3
4	AKT1	3O96	Co-Crystal	−14.9
			Etoposide	−13
			Ponatinib	−12.6
			Imatinib	−11.5
			Novobiocin	−11.5
			Daunorubicin	−9
5	STAT3	6NJS	Pimozide	−7.5
			Alectinib	−7.1
			Rapamycin	−7.1
			Sorafenib	−6.9
			Dasatinib	−6.7
			Co-Crystal	−6.7

## Data Availability

The protein structures used in this investigation were retrieved from the RCSB Protein Data Bank (https://www.rcsb.org/) with the PDB IDs: 2AZ5, 1ALU, 1ITB, 3O96, 6NJS. Ligand structures were obtained from the PubChem database (https://pubchem.ncbi.nlm.nih.gov/). Targets associated with rheumatoid arthritis (RA) were collected from the DisGeNet webserver (https://www.disgenet.org/). Protein–protein interaction (PPI) and protein–drug interaction (PDI) networks were generated using Cytoscape 3.10.1 and its STRING, STITCH, and CytoNCA plugins. Ligand preparation was carried out using Marvin Sketch, while protein preparation was conducted using Swiss-PDB Viewer. Molecular docking simulations were performed using PyRx 0.8, employing AutoDock Vina. Visualization of protein–ligand interactions was performed using Discovery Studio Visualizer. Molecular dynamics (MD) simulations were conducted using the Desmond module of the Schrödinger Suite 2021-4.

## Abbreviations

ACPA	Anti-Citrullinated Protein Antibodies
AKT1	AKT Serine/Threonine Kinase 1
CIA	Collagen-Induced Arthritis
DMARDs	Disease-Modifying Anti-Rheumatic Drugs
FDA	Food and Drug Administration
HLA-DRB1	Human Leukocyte Antigen-DRB1
IL-1β	Interleukin-1beta
IL-6	Interleukin-6
MD	Molecular Dynamics
MeSH	Medical Subject Headings
NSAIDs	Non-Steroidal Anti-Inflammatory Drugs
NPT	Isothermal–Isobaric Ensemble
OMIM	Online Mendelian Inheritance in Man
PDB	Protein Data Bank
PDI	Protein–drug Interaction
PLIP	Protein–Ligand Interaction Profiler
PPI	Protein–protein Interaction
RA	Rheumatoid Arthritis
RA-FLS	Rheumatoid Arthritis Fibroblast-Like Synoviocytes
RESPA	Reversible Reference System Propagator Algorithm
RMSD	Root Mean Square Deviation
RMSF	Root Mean Square Fluctuation
SPME	Smooth Particle Mesh Ewald
STAT3	Signal Transducer and Activator of Transcription 3
TNF-α	Tumor Necrosis Factor-alpha
UMLS CUI	Unified Medical Language System Concept Unique Identifier
